# Aphallia: Report of three cases and literature review

**Published:** 2016-04

**Authors:** Fereshteh Talebpour Amiri, Davood Nasiry Zarrin Ghabaee, Ramezan Ali Naeimi, Seyed Javad Seyedi, Seyed Abdollah Mousavi

**Affiliations:** 1 *Department of Anatomy, Faculty of Medicine, Mazandaran University of Medical Sciences, Sari, Iran.*; 2 *Student Research Committee, Mazandaran University of Medical Sciences, Sari, Iran.*; 3 *Shahid Beheshti University, Tehran, Iran.*; 4 *Department of Surgery, Faculty of Medicine, Antibacterial Resistance Research Center, Mazandaran University of Medical Sciences, Sari, Iran.*

**Keywords:** *Aphallia*, *Development of phallus*, *Associated anomalies*

## Abstract

**Background:**

Aphallia or penile agenesis is a rare malformation accompanying with no phallus. This anomaly is extremely rare with abnormality of urogenital system and psychological consequences. Its outbreak is estimated 1 out of 10-30 million births.

**Case:**

Reviewing 3 cases of male external genitalia agenesis, which associated with multiple anomalies of musculoskeletal, cardiovascular and genitourinary system.

**Conclusion:**

Aphallia has psychosocial consequences and a guarded prognosis. This study showed that if the kidney failure is due to its obstruction, these patients will be born in more favorable conditions and the future treatment measures will be directed to keep the external genitalia (male) through timely diagnosis and prenatal surgery and timely bladder drainage.

## Introduction

Aphallia or complete penis agenesis is a very infrequent congenital abnormality with dramatic psychological consequences (1). Its prevalence is estimated one per 10-30 million births (2-5). The reason behind it is genital tubercle development. This anomaly has often been reported with abnormalities like Kidney agenesis or cystic kidney, horseshoe kidney, urinary reflux, prostate agenesis, skeletal and neural disorders, annular pancreas, clubfoot and heart problems (6, 7). 

Since the digestive system and urinary tract caudal embryonic origin are of cloaca segment, recto urethral fistula is seen in majority of these patients. The majority of such patients have 46XY Karyotype. Because of phallus not being formed despite male genotype, many of these people suffer from numerous psychological complications. Herein we report three cases of Aphallia along with a review of literature.

A signed informed consent was obtained from parents of all patients who participated in the study.

## Case Reports

**Case 1**

A baby was born weighing 2 kg with 45 cm height at week 31 due to amnion getting torn via caesarean section (CSC) from a 31-year-old mother with no prior sonography before birth as her second delivery. Her first baby was a healthy girl. She had a previous record of unknown abortion and during recent pregnancy, she took progesterone vaginal suppositories to avoid abortion recurrence. There was no parental family relation, no pointed congenital anomaly in their family history and not taking any other drugs. 

**Figure 1:**
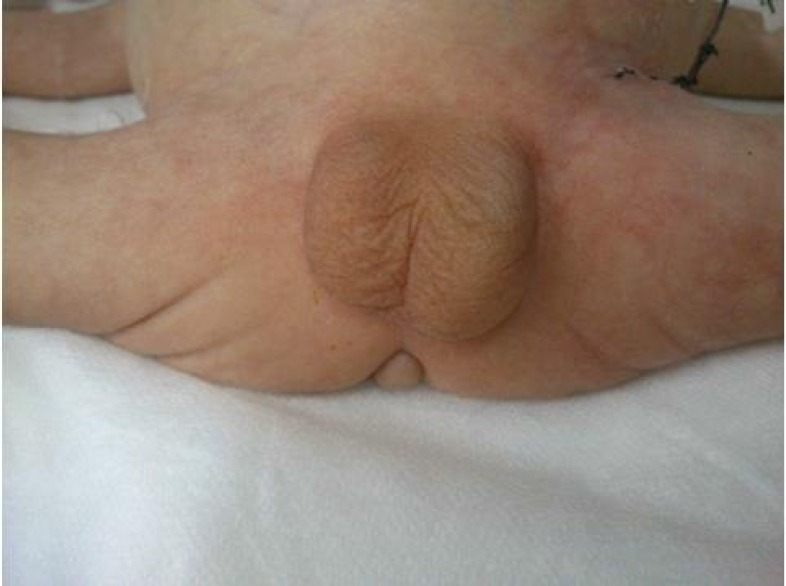
Aphallia with Skin tag

No phallus has been seen in baby via clinical examination. Scrotum wrinkle and testicular descent was revealed normal and Male gender was verified by karyotyping (Figure 1). Examining perineum, anterior skin tag to anus was observed. Anus was in normal position. Urinary opening wasn’t seen in perineum. Urine was mixed with meconium discharged into dentate line proximity via urinary endoscopic examination. Examining hip, there was limitation in hip abduction. Echocardiography and ultrasonography of brain were normal.

In abdominal and pelvis ultrasonography, severe hydronephrosis and significant diminished left renal cortical thickness along with tough dilation and cystic pelvis were spotted (Figure 1). Mild to average hydronephrosis was seen in the right kidney with mild reduced cortical thickness and pelvis dilatation. In rectum-injected contrast media cystogram, left vesicoureteral reflux was observed and urethro- rectal fistula was verified. Cystostomy was done the day after birth.

The patient was discharged for penis reconstruction treatment plan.

**Figure 2:**
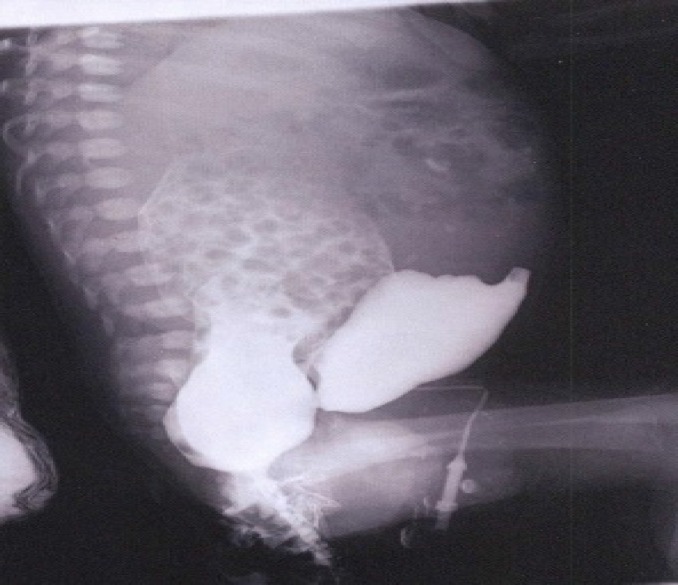
Cystogram represents a Uretrorectal fistula

**Case 2**

A premature neonate (gestational age 32-week) weighing 1800 gr with no penis was born from a 27 yrs old mother with no special disease and a healthy boy. The parents were healthy with no family relation. No drug was taken during gestation and no certain chemical proximity was noted. The mother had once unknown induced abortion history. The only mentioned point in pregnancy period sonography has been Oligopolyhydramnios. 

**Figure 3:**
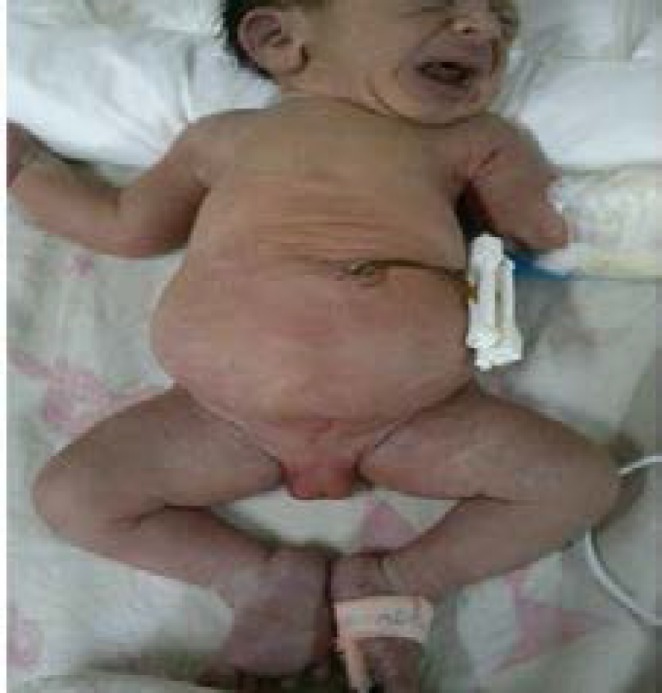
Aphallia with Abdominal distention, Position the top navel and clubfoot

The baby's Karyotyping was XY. Examination revealed his respiratory distress. Checking his head and neck, the ears were located lower than their normal position and saddle nose was observed. There were featured chest, distended belly and higher located navel and the liver was touched 3 cm below the ribs. Via examining genitals, phallus has not been seen. Scrotum was normal and contained gonad with normal dimensions and consistency. Sacrum was short but anus was normal with normal meconium excretion. In the lower limbs, two sided clubfoot was observed (Figure 3). 

**Figure 4:**
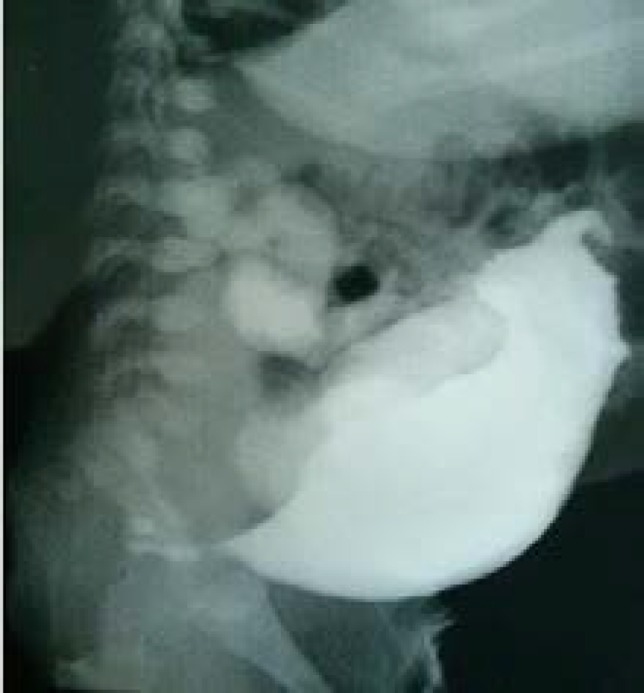
Cystogram showed severe dilation of the bladder and ureter bilateral reflux and rectal fistula

Echocardiography reported mild tricuspid heart insufficiency and small openness of patent ducts arteriosus. In abdominal sonography, massive bladder with increased thick wall has been seen. The kidneys were severely atrophic with several cysts (multicystic bladder). In cystography, the contrast media reflux into urachus and bilateral ureter was observed. Urethra was short leading to distal rectum but no obvious opening was spotted (Figure 4).

Due to no urination, the patients underwent cystostomy. During the operation, biopsy was taken from gonads, which reported normal testes. 2^nd^ day after birth, blood urea, creatinine and potassium increased and on the 25^th^ postnatal day, the baby died because of clinical signs of renal failure.

**Case 3**

**Figure 5:**
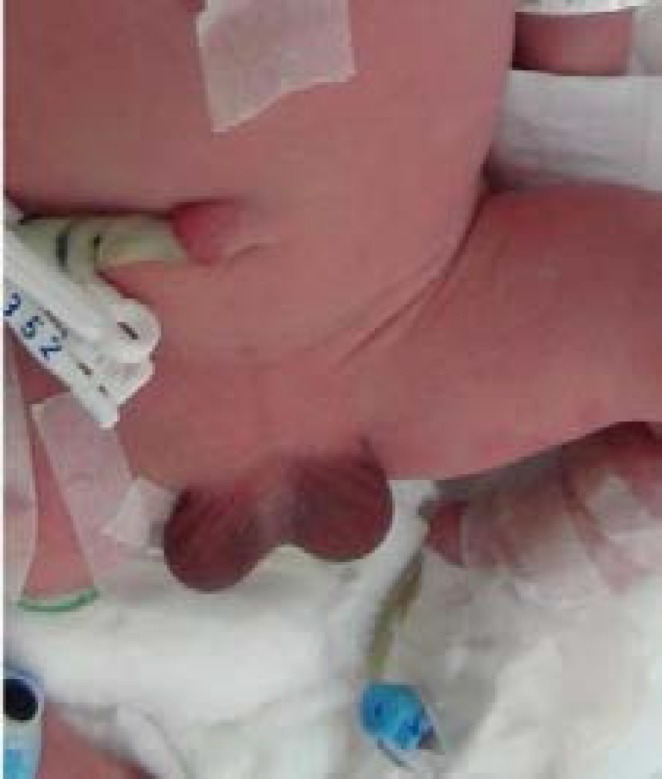
Aphallia with bifid scrotum

A neonate (2 kg weight and 44 cm height) was born in the 37^th^ week of pregnancy via caesarean section as one of the twins that the other one had no problems. There was no family history between the parents. The mother was 39 years old who took 100 mg aspirin daily for 8 months. Examination revealed aphallia accompanied with bifid scrotum containing gonad (Figure 5). Cardiography reported mild tricuspid valve failure. Ultrasonography of brain was normal. Kidneys and bladder sonography reported no pathological problem. Urination was seen from a small urethra below and anal verge anterior and was catheterized. Cystogram showed mild left side reflux. Since the parents didn’t agree with the subsequent follow-up, the baby was discharged because of his parents' consent.

## Discussion

Aphallia or agenesis phallus is a congenital disorder that had been reported fewer than 100 cases until 2010 worldwide (1, 8). This anomaly occurs within 4 wks of embryonic development accompanying other anomalies (1, 9). The reason behind it is associated with no genital tubercle formation or its development impairment (6, 7). Genital tubercle, embryonic progenitor mesenchymal tissue in man and clitoris in woman, is formed through tissue budding mesenchymal tissue underneath genital ectoderm next to cloacal membrane where mesenchymal reproduction impairment results in Aphallia (6, 7). The anterior and cranial mesoderm (cephalic segment) from urogenital sinus plays its role by expressing Bone Morphogenetic Protein 4 (BMP4) Factor in genital tubercle formation and ultimately phallus formation. Congenital anomalies accompany wide defects in blastogenesis under this certain time limit (10). 

Gerard-Blanluet reported Aphallia with several mesodermal impairments in 2011. This baby was born with closed anus, dysplasia of both kidneys, complete right lung agenesis and rib segmental disorder (9). In 1999, Gripp issued a report about a case of Aphallia with anal stenosis, phallus tetralogy, and multi spinal disorders such as sacrum agenesis and central nervous system (CNS) (11). In our patients, renal hydronephrosis, multicystic kidney and short sacrum have been reported, too. Newly born babies with Aphallia have Karyotype XY46. *SRY* gene is the sex determining transcription factor on Y chromosome. This gene expression leads to gonad development as male gender and Anti-Mullerian Hormone is secreted from developing testicle's Sertoli cells so that internal and external genital system is inclined to male gender (10). 

In addition to *SRY* gene, external genital system develops as cauda from septum whose development basically depends on androgen (testosterone) and similar to other septum organs, epithelial-mesenchymal interaction plays the key role in their development (12). In 2011, Wang introduced a case of Aphallia with 46 XY chromosome Karyotype, where PCR test revealed no azoospermia factor or *SRY* gene (5). Urorectal Septum Malformation Sequence (URSMS) is a rare syndrome whose pathogenic mechanism lies in its defect related to urorectal septum and cloacal segment division and or its cloacal membrane binding. Cloacal membrane remaining results in abnormal development of perineum holes and rectourethral fistula (13). 

This syndrome is mesodermal disorder reported with several anomalies including anal stenosis or closed anus, renal dysplasia, lung agenesis, phallus tetralogy, multi spinal disorders like sacrum agenesis, rib segmental and CNS disorders (11). Besides these disorders, Aphallia has also been reported in some cases (9, 14). Urorectal septum divides cloacal area into two completely separate parts of urogenital sinus and anorectal canal. If urorectal septum caudal segment doesn’t develop completely, it leads to rectourethral fistulas. In men, this binding usually forms rectourethral fistulas (8). Aphallia with rectourethral fistula has been reported by authors majority. In all three mentioned cases Aphallia with rectourethral fistula has been reported. The outbreak of urorectal septum development is 1 per 5000 births (10). 

In patients with Aphallia, urine usually passes from front part of rectum. This anomaly usually accompanies anteposed anus, vesicoureteral reflux and other disorders (8). Examining 50 patients, Skoog and Belman classified phallus agenesis into three types as presphictric, postsphictric and urethral atresia regarding urethral opening position (15). Due to suffering from several disorders, many patients die a few days after birth. This is seen at higher level when urethral opening is in higher parts of rectum (12). In our report the baby whose fistula opened to rectum died after 7 days of birth. So far, various disorders have been reported along with Aphallia. So that 54% of cases accompanied genitourinary anomaly like renal agenesis or cystic kidney, horseshoe kidney, urinary reflux and prostate agenesis. Of the other anomalies, we can point out sexual and neurological disorders, annular pancreas, clubfoot or heart problems (4) 

Aphallia has been reported among diabetic mothers (4, 11). But urogenital system abnormalities are more prevalent among controlled diabetic mothers (11). In the majority of patients suffering Aphallia, renal agenesis and urinary tract bring about fatal oligohydraminos and subsequently, pulmonary hyperplasia (10). Benedetto *et al* reported one case of Aphallia in monoamniotic twins with scrotum and two normal gonads and no bladder, urinary tract or kidneys(11). Cloacal anomalies are also related to anorectal segment anomalies reported in monoamniotic twins (14). Low amniotic volume was seen in babies with lung development anomaly (10, 14).

In one of present reported cases, the mother had one abortion and used pregnancy suppositories. Maybe role of progesterone in this anomaly during gestation requires further investigation. In one of referred patients, besides heart, kidney, and skeletal problems, hepatomegaly and dropped ears are the common symptoms of this disease not mentioned so far in papers. Also about the third reported case, bifid scrotum wasn’t observed and hasn’t been pointed out yet. As differential diagnosis, Aphallia should be distinguished from anomalies such as severe microphallus, epispadias or hypospadias and intrauterine penis amputation. 

In these patients in cases of surviving in later months, their genitals reconstruction is taken into account. Although in the past, transgender to female was more preferred due to operation ease, today because of future psychological traumas, efforts are made to reconstruct genitals for male gender (15, 16). In cases when we are going to do transgender, it is suggested to do bilateral gonadectomy early days after birth and in puberty, estrogen therapy and vaginoplasty for breast and the other gender features development in women are suggested. Separating urinary system from digestive system has to be done as soon as possible (4). 

If the child is going to stay boy as his parents decide, phalloplasty has to be conducted at puberty time. Thanks to IVF advances, having a child will be possible for these patients. Unfortunately, one of our cases died three weeks after birth, the reason behind which was kidney failure like similar cases manifested itself as postnatal oligohydraminos. Overall, based on novel treatment measures, if the kidney failure is due to its obstruction, these patients will be born in more favorable conditions and future treatment measures will be directed to keep external genitals (male) through timely diagnosis and prenatal surgery and timely bladder drainage (fetal surgery). 

## Conflict of interest

The authors have no conflicts of interest.
